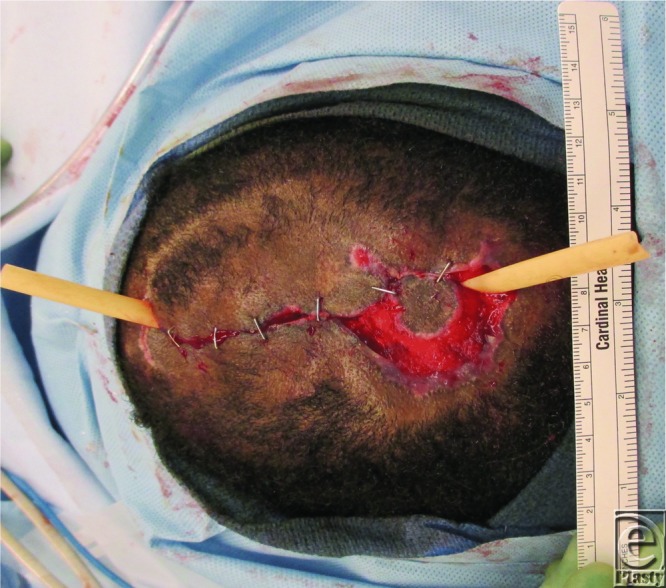# Hair Braiding-Induced Scalp Necrosis: A Case Report

**Published:** 2016-04-25

**Authors:** Zachary Borab, Madeleine Gantz, Michael Mirmanesh, Hengli Lin

**Affiliations:** ^a^Drexel University College of Medicine, Philadelphia, Pa; ^b^Division of Plastic Surgery, University of California Davis, Sacramento, CA; ^c^Department of Plastic Surgery, Kaiser Permanente South Sacramento, Sacramento, CA

**Keywords:** traction-induced scalp necrosis, scalp necrosis, traction alopecia, hair braiding scalp necrosis, hair braiding

Initially, the patient's scalp was shaved to expose the entire lesion (Fig 1) for thorough irrigation, debridement, and introduction of wet to dry dressings. She was discharged on empiric cephalexin. Seven days after initial presentation (Fig 2), she was taken to the operating room for further debridement, washout, and local tissue rearrangement. Dense amounts of necrotic tissue were debrided, extending through the subcutaneous tissue, exposing some areas of galea. Scalp laxity and undermining allowed significant advancement and primary closure, leaving a markedly diminished wound (Fig 3). After a clean, healthy wound bed was ensured postoperatively, local wound care was instituted. At her 6-month follow-up, the patient's wound was healed with obvious areas of scar alopecia. The remaining scar tissue was excised and scalp flaps were advanced, eliminating any remaining areas of alopecia.

## DESCRIPTION

An 18-year-old African American woman with a history of sickle cell trait presented with 3 weeks of foul-smelling discharge from her scalp. Physical examination revealed a 10 × 20-cm irregular and weepy scalp defect at various stages of healing.

## QUESTIONS

1. **How common is scalp necrosis from hair braiding?**
2. **What are the other known complications of hair braiding?**
3. **What are the potential causes of scalp necrosis?**
4. **How should scalp necrosis be managed?**

## DISCUSSION

There is only one previously reported case of scalp necrosis induced by hair braiding, which details a 41-year-old African American woman who experienced swelling and tenderness in the parietal region of her scalp after tight hair braiding. A few days later, the area became ulcerated, resulting in a 10 × 7-cm wound with a rim of alopecia. She was treated with hydrocolloid gel dressing, followed by regular dressings. Permanent hair loss was minimal, and no surgery was indicated.^1^

Known complications of hair braiding include scalp necrosis, subgaleal hematoma, and traction alopecia.^1^^-^3 Interestingly, subgaleal hematomas have been reported as a primary manifestation of familial bleeding disorders such as von Willebrand disease and factor XIII deficiency.^2^^,^^3^ Traction alopecia describes hair loss secondary to chronic tension on hair follicles and is the most common complication of braided hairstyling.^1^ Some examples of at risk areas include the frontal scalp and submandibular area in Sikh males, the occipital region in ballerinas, and the parieto-occipital scalp in nurses wearing nursing caps. A common feature in all of these cases is that the area of alopecia corresponds with the site(s) under greatest tension for that particular hairstyle. The likely cause for scalp necrosis in our case is traction induced. The history of tight hair braiding in conjunction with an area of alopecia surrounding the scalp wound on physical examination supports this diagnosis.

Scalp necrosis is a complication most commonly associated with giant cell arteritis, malignancy, and radiotherapy.^4^ Cases of scalp necrosis associated with other clinical entities are scarce in the literature. Sickle cell trait has not been associated with hair braiding or scalp necrosis. Unlu and de Vries^5^ published a case of ischemic scalp ulceration and hair loss due to insufficient tissue perfusion secondary to atherosclerosis which improved following revascularization. Another group reported 2 cases of scalp necrosis following preoperative embolization for meningeal tumors.^6^

Selection of a technique that can safely restore aesthetic features of the scalp while permitting the scalp to perform its protective and functional duties is the ultimate goal of reconstruction. Healing by secondary intention is appropriate for a small defect with a healthy wound base. This method of healing can leave the patient with alopecia that will either to be addressed once healing is complete or masked with routine hairstyling practices. Skin grafting with or without available dermal matrices can allow for coverage of exposed tissue for a patient with scarce local tissue rearrangement options. It is a quick and technically easy procedure, but it does not adequately address contour or hair loss deformities, and carries with it varied donor site morbidity, depending on the size of the defect. Primary closure and local-regional flaps limit alopecia and contour deformities, and they may increase the likelihood of reconstruction with a one-step procedure. Disadvantages include the need for extensive undermining or relaxing incisions that may alter the hairline. There are a number of local flaps available in reconstruction. Use of large rotational advancement flaps for scalp defects helps compensate for the relative inelasticity of the scalp tissue and can carry adequate blood supply. Incision orientation is made with both preservation of the hairline and inclusion of a single scalp pedicle in mind. Tissue expansion is a valuable tool when attempting to reconstruct larger wounds. It does, however, require multiple procedures, carries an increased risk of infection, and is associated with complications in the irradiated patient.^7^ In our case, a combination approach including healing by secondary intention, local tissue advancement, and scar revision was used to obtain an acceptable aesthetic outcome for the patient.

## Figures and Tables

**Figure 1:**
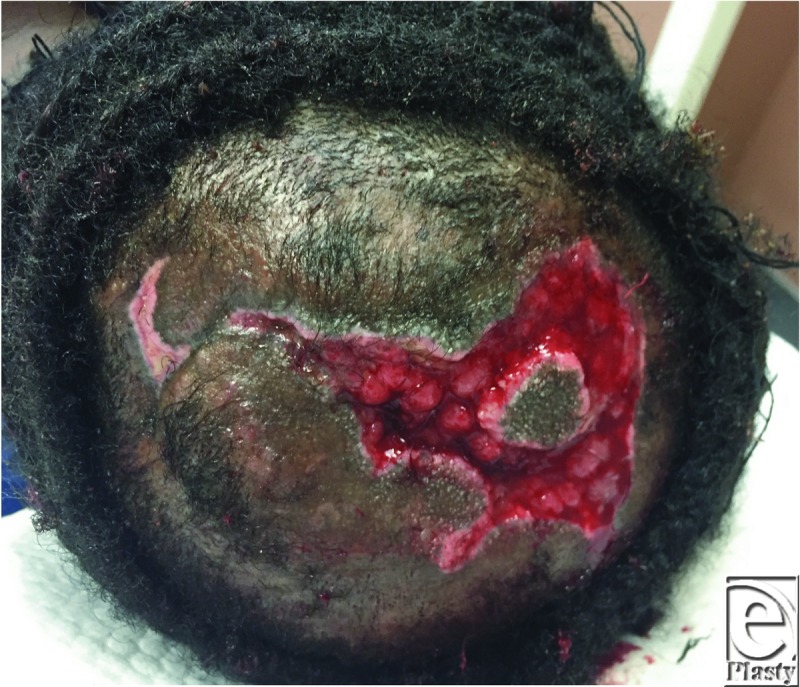


**Figure 2:**
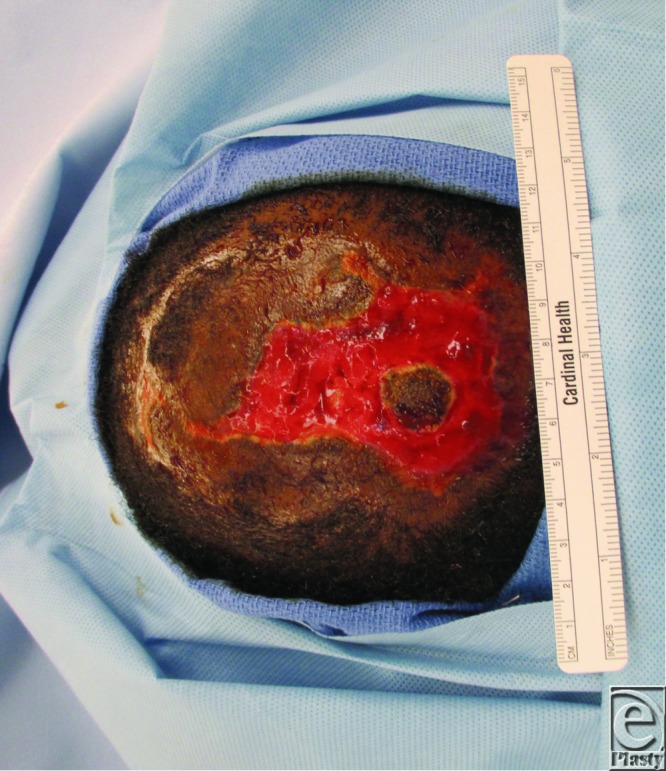


**Figure 3:**